# A Case of Reverse Takotsubo Cardiomyopathy Incited by a Spinal Subdural Hematoma

**DOI:** 10.1155/2019/9285460

**Published:** 2019-07-22

**Authors:** Kyle Sanchez, Steven Glener, Nathan E. Esplin, Okorie N. Okorie, Amay Parikh

**Affiliations:** ^1^University of Central Florida College of Medicine, 6850 Lake Nona Boulevard, Orlando, FL 32827, USA; ^2^Department of Neurosurgery, Allegheny General Hospital, 320 E North Ave., Pittsburgh, PA 15212, USA; ^3^Florida Hospital, 601 E Rollins Street, Orlando, FL 32803, USA

## Abstract

Takotsubo cardiomyopathy is a rare syndrome of transient, reversible left ventricular systolic dysfunction. It mimics myocardial infarction clinically and includes elevated cardiac enzymes, but echocardiography reveals apical ballooning and basal hyperkinesis. Infrequently, midventricular or even reverse Takotsubo patterns have been described, involving ballooning of the basal heart without the characteristic ‘Takotsubo' appearance. There are cases in the literature that support a connection between reverse Takotsubo cardiomyopathy (r-TTC) and neurological insults as inciting factors. We report a case of r-TTC in an otherwise healthy 23-year-old man presenting with back pain, urinary retention, bradycardia, and hypertension. Troponin levels and brain natriuretic peptide (BNP) were elevated, and echocardiogram revealed an ejection fraction (EF) of less than 20%. In addition, MRI demonstrated a spinal subdural hematoma from T1-S1 with no cord compression. Repeated echocardiogram demonstrated an EF of 20-25% with a reverse Takotsubo pattern of cardiomyopathy. With supportive care, his clinical picture improved with normalization of cardiac enzyme and BNP values. This case represents a r-TTC presenting as heart failure in a young, apparently healthy male likely incited by a spinal subdural hematoma. To our knowledge, it is the first of its kind reported.

## 1. Introduction

Takotsubo Cardiomyopathy (TTC), also called stress-induced cardiomyopathy or broken heart syndrome, is a rare syndrome of transient and reversible left ventricular systolic dysfunction in the absence of coronary stenosis. TTC is characterized by ballooning of the left ventricular apex that is provoked by an emotionally or physically stressful experience [1], although the mechanism is still debated. The clinical presentation of TTC mimics acute coronary syndrome, as it presents with symptoms ranging from isolated chest pain to severe dyspnea and cardiogenic shock, often accompanied by elevation of troponin level and T-wave and ST-segment abnormalities on echocardiogram (ECG) [2]. Classically, echocardiogram shows apical ballooning and basal hyperkinesis, resembling a ‘crab pot' or ‘octopus trap' that, from the Japanese word for such, gives this disorder its name.

The four known patterns of left ventricular involvement in TTC include classical type, reverse type, midventricular type, and localized type [2]. In the classical pattern of TTC, the most common type, the base of the ventricle contracts normally or is hyperkinetic while the rest of the ventricle is dyskinetic or akinetic [3], causing the classical ballooning of the left ventricular apex. Reverse, or inverted type, Takotsubo cardiomyopathy (r-TTC) is a rare variant characterized by hypokinesis of the base with relative sparing of the midventricle and apex [1], causing ballooning to occur at the base of the heart.

In comparison to other patterns of TCC, r-TCC usually presents in a younger patient population with higher troponin levels, lower ejection fraction, and a paradoxically faster recovery [4]. The higher troponin levels are believed to be the result of a larger region of myocardial involvement in r-TTC in comparison to the apex alone [2]. On the contrary, natriuretic peptides are often more elevated in apical and midventricular patterns of TCC and account for the higher prevalence of severe symptoms and higher NYHA functional class [2] seen in comparison to r-TTC. The higher prevalence of symptoms despite less damage to cardiomyocytes may be explained by the frequent coexistence of mitral regurgitation caused by apical ballooning [2]. Predictors of mortality from r-TTC include decreased left ventricular ejection fraction, development of atrial fibrillation, and neurologic disease.”

The precise etiology of TCC/r-TCC is unknown, but evidence indicates that both patterns are associated with strong surges of adrenergic outflow that occur following intense emotional or physical stress [5–9], where high circulating levels of epinephrine may cause coronary spasm, endothelial dysfunction, and indirect myocardial damage [2]. A few case studies have suggested a potential connection between r-TTC and neurological insults as inciting factors. Reported neurological stressors which have induced r-TCC include cerebellar hemorrhage [2], medulla oblongata hemorrhage [5], subarachnoid hemorrhage [10], inadvertent intrathecal injection [11], and multiple sclerosis [12]. Additionally, the presence of neurologic disease may be a good predictor of mortality in patients with r-TTC [13]. To the best of our knowledge, this is the first report of a case of r-TTC presenting after a spinal subdural hematoma.

## 2. Case Presentation

A 23-year-old man presented to an outside Emergency Department (ED) with difficulty in voiding both bowel and bladder, and significant mid-back pain with associated weakness, numbness, and tingling in bilateral lower extremities. He reported a two-week history of headache, nonproductive cough, nasal congestion, nausea, and vomiting but denied flank pain and dysuria. On physical exam, he was significantly hypertensive (225/119 mmHg) and bradycardic (57 bpm), with paraspinal muscle tenderness, but there was no vertebral point tenderness. He was also found to have hypoactive deep tendon reflexes at the patella and Achilles tendons.

He had visited the ED twice in the two days prior to this admission for constant, “achy” back pain in the lumbar region, exacerbated by movement, which he attributed to lifting boxes at work. During these hospital visits, the patient denied bowel and bladder dysfunction, saddle anesthesia, fever, and chills. On both occasions, he was discharged home with the diagnosis of acute lumbar paraspinal muscle strain.

On this third visit, now with urinary retention, he was admitted to the outside facility. The patient was catheterized, yielding 1400 mL of urine, which tested positive for THC and oxycodone and revealed trace amounts of ketones. Initial laboratory evaluation was significant for leukocytosis (14,580 cells/*μ*L, neutrophil 81.6%) and elevated N-terminal BNP at 734 pg/mL (0 – 125 pg/mL).

**Figure 1:**
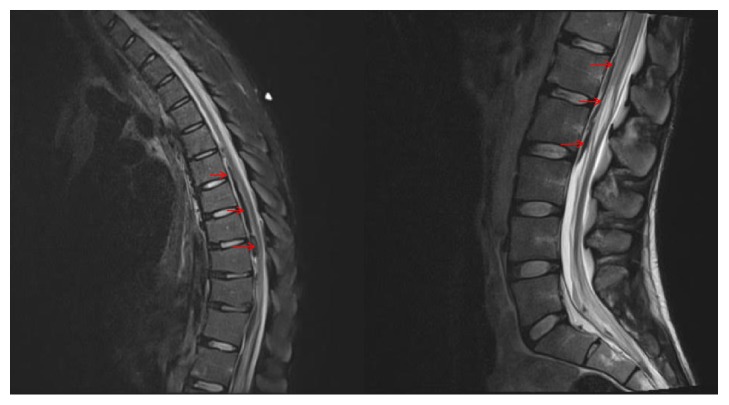
MRI showing thoracic (*left*) and lumbar (*right*) subdural hematomas (arrows).

Computed tomography (CT) of the head showed no evidence of significant intracranial pathology. CT of the abdomen and pelvis showed no acute findings with no evidence of intra-abdominal trauma. Computed tomography angiogram (CTA) of the chest revealed symmetric infiltrates and interlobular septal thickenings of the lungs, presumed to be pulmonary edema, although diffuse multifocal pneumonia and pneumocystis pneumonia were considered. It also revealed dilation of left ventricle and left atrium, pneumomediastinum, and fluid in the esophagus. MRI of the lumbar spine revealed abnormally decreased T2 signal and mildly increased T1 signal involving the ventral central canal spanning from T5 to L2-L3, which likely reflected a subdural hematoma (Figure 1). It also demonstrated a likely superimposed subdural hygroma with fluid signal present that was most prominent beginning at the L2-L3 level and progressing caudally to the L5-S1 level within the ventral thecal sac. There was associated dorsal displacement of the nerve roots of the cauda equina and crowding of the nerve roots. These findings resulted in diffuse narrowing of the lumbar thecal sac most severely between T7-T8 and the mid-T10 vertebral body, where the subdural hematoma was largest. Narrowing of the thecal sac was most severe at the T9-T10 level where there was moderate to severe narrowing of the thecal sac present with deformation of the anterior surface of the cord that was worse on the left. Small active Schmorl's nodes were demonstrated along the inferior endplate of L1. There was also an abnormal signal within the subarachnoid spaces of the lower lumbar spine that was most prominent at the L5-S1 level. The decreased T2 signal and increased T1 signal present at this level likely reflected a subarachnoid hemorrhage. There was no enhancement on the study to suggest infection and no fracture or subluxation. After experiencing a hypoxic event with O2 saturation dropping to 70%, he was then transferred to our tertiary care center.

On admission, physical exam revealed full strength in upper and lower extremities with normal light touch sensation. However, he reported quadricep and calf numbness, despite the normal light touch sensation. Laboratory evaluation was significant for leukocytosis (20,970 cells/*μ*L) (neutrophil 77.1%), elevated N-terminal BNP level (4,325 pg/mL), and troponin T level elevation at 0.6 ng/mL (0 – 0.14 ng/mL).

Repeated noncontrast MRI of the thoracic spine ordered by neurosurgery showed acute subdural hematoma involving the thoracic spine and the ventral central canal that was worse on the left, spanning from the T5 level caudally to involve the lumbar spine. Despite prior suspicion of increased T2 signal involving the ventral cord spanning the T7-T8 level to mid-T10 vertebral body, evaluation for cord signal abnormality was deemed severely limited secondary to motion artifact. There were small areas of acute subdural hemorrhage within the dorsal aspect of the thecal sac most prominent at the T4-T5 level with suspicion for a small amount of dorsal subdural hygroma involving the thoracic spine (Figure 1). No fracture or subluxation was seen. It also demonstrated airspace consolidation involving the bilateral lung bases and the dependent portion of the right upper lobe of the lung. These findings were believed to have reflected severe pulmonary edema. CT of the head without contrast was negative for large territorial acute infarction, acute intracranial hemorrhage, and mass effect. CT angiogram of the head showed no evidence of significant intracranial arterial pathology. Repeated MRI of the lumbar spine showed loss of T2 prolongation in the ventral subdural region from the level of L1-L3, which was originally felt to represent a flow-related artifact. Upon further review, this may have represented a true fluid collection. From the level of L3-L5, there may have been a possible subdural hygroma (Figure 1). However, the etiology of subdural hematoma was unclear; infectious or traumatic etiologies were suspected.

ECG showed sinus bradycardia with possible left atrial enlargement and left ventricular hypertrophy. Echocardiogram revealed an ejection fraction of 20-25%. The etiology of cardiomyopathy was also unclear, but viral infection was suspected.

**Figure 2:**
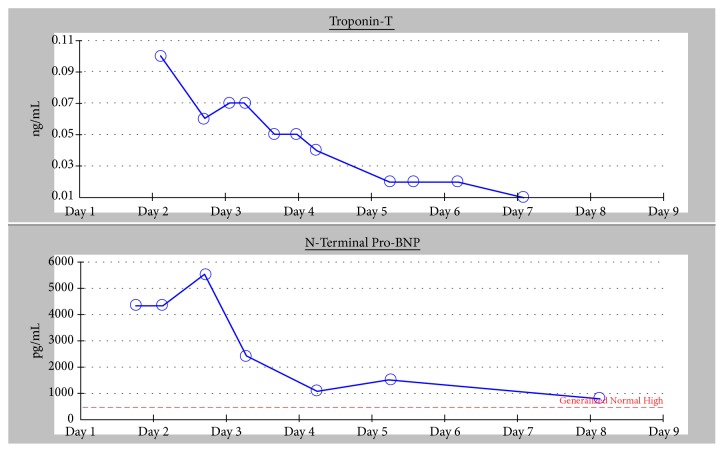
Troponin-T and N-Terminal Pro-BNP levels over the course of hospital stay.

Antibody testing was conducted for HIV,* Toxoplasma *spp., and EBV; PCR testing was conducted for HHV-6 and Parvovirus B19; serological testing was conducted for Coxsackieviruses A and B; and blood cultures were taken, all of which were negative. During his stay, the patient's N-terminal BNP level steadily declined after peaking at 5,516 pg/mL, as did his troponin T levels (Figure 2). A repeated echocardiogram on hospital day six revealed an ejection fraction of 45-50%, with normal left ventricular size, mild right ventricular dilation, mild mitral regurgitation, trace tricuspid regurgitation, and normal systolic function. An additional echocardiogram the following day showed an ejection fraction of 55-60% with mild mitral regurgitation, trace tricuspid regurgitation, and greater than 50% respiratory change in the inferior vena cava dimension from normally sized chambers.

The patient was diagnosed with a case of likely reverse Takotsubo cardiomyopathy potentially secondary to subdural hematoma of the thoracic and lumbar spine.

## 3. Discussion

We believe this to be a case of r-TTC presenting as heart failure in a young, apparently healthy man likely incited by a spinal subdural hematoma.

**Figure 3:**
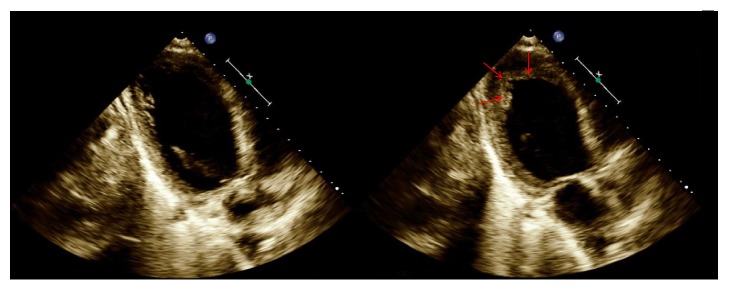
TTE apical two-chamber view of left ventricle in End Diastole (*left*) and End Systole (*right*), demonstrating poor ventricular compression and low EF. r-TTC pattern is indicated by contraction of ventricular apex (arrows) and hypokinesis of base during systole.

TTC is a rare syndrome of transient, reversible left ventricular dysfunction that is provoked by physical or emotional stressors [1]. Of the four types of TTC, the hallmark of r-TTC is the presence of an inverted Takotsubo pattern with hypokinesis of the left ventricular base and sparing of the apex, causing ballooning of the base rather than apex, which was observed on the patient's echocardiogram (Figure 3). All patterns of TTC typically mimic acute coronary syndrome and related cardiovascular complications [1], but r-TTC is associated with higher troponin levels and lower EF [2]. In this patient, we believe that r-TTC explains the heart failure with reduced EF. The transient nature of the patient's condition, as evidenced by the resolution of symptoms and normalization of Troponin-T levels, N-terminal BNP levels, and EF, is consistent with the spontaneous resolution of all cases of TTC, especially those of r-TTC [2]. In addition, the patient's young age is typical of r-TTC [2, 14].

While all patterns of Takotsubo cardiomyopathy are typically preceded by emotional or physical stressors [1], the patient's history did not indicate that he experienced any intense stressor before his condition developed. We recognize that this is atypical, even for r-TTC. However, there have been multiple cases that suggest a potential connection between r-TTC and neurological insults as inciting factors [4–12]. The patient initially presented with back pain, which was likely the result of his underlying subdural hematoma. Etiology of the subdural hematoma was unclear, and infectious or injury-induced causes were suspected. However, infectious workup was negative, and patient denied any overt injury aside from his back pain. Regardless of the etiology of his subdural hematoma, we believe that it then incited a r-TTC presenting as heart failure with reduced EF.

To our knowledge, this is the first case reported of a spinal subdural hematoma inciting r-TTC. It demonstrates the critical nature of this rare presentation of poorly understood pathology and drives home the need to keep it on the differential in a case of cardiomyopathy or heart failure.
